# Antioxidant Capacity, Cytotoxicity, and Acute Oral Toxicity of *Gynura bicolor*

**DOI:** 10.1155/2013/958407

**Published:** 2013-12-04

**Authors:** Wuen Yew Teoh, Kae Shin Sim, Jaime Stella Moses Richardson, Norhanom Abdul Wahab, See Ziau Hoe

**Affiliations:** ^1^Institute of Biological Sciences, Faculty of Science, University of Malaya, 50603 Kuala Lumpur, Malaysia; ^2^Biology Division, Centre for Foundation Studies in Science, University of Malaya, 50603 Kuala Lumpur, Malaysia; ^3^Department of Physiology, Faculty of Medicine, University of Malaya, 50603 Kuala Lumpur, Malaysia

## Abstract

*Gynura bicolor* (Compositae) which is widely used by the locals as natural remedies in folk medicine has limited scientific studies to ensure its efficacy and nontoxicity. The current study reports the total phenolic content, antioxidant capacity, cytotoxicity, and acute oral toxicity of crude methanol and its fractionated extracts (hexane, ethyl acetate, and water) of *G. bicolor *leaves. Five human colon cancer cell lines (HT-29, HCT-15, SW480, Caco-2, and HCT 116), one human breast adenocarcinoma cell line (MCF7), and one human normal colon cell line (CCD-18Co) were used to evaluate the cytotoxicity of *G. bicolor*. The present findings had clearly demonstrated that ethyl acetate extract of *G. bicolor *with the highest total phenolic content among the extracts showed the strongest antioxidant activity (DPPH radical scavenging assay and metal chelating assay), possessed cytotoxicity, and induced apoptotic and necrotic cell death, especially towards the HCT 116 and HCT-15 colon cancer cells. The acute oral toxicity study indicated that methanol extract of *G. bicolor* has negligible level of toxicity when administered orally and has been regarded as safe in experimental rats. The findings of the current study clearly established the chemoprevention potential of *G. bicolor *and thus provide scientific validation on the therapeutic claims of *G. bicolor*.

## 1. Introduction 

Plants have been used to maintain human health for a very long time. It is well known that some of the plant species possess interesting bioactivities with potential therapeutic applications which can benefit us. *Gynura bicolor*, locally known as “Hong Feng Cai” (Chinese) and “Sambung Nyawa Ungu” (Malay), belongs to the botanical family of Compositae. The leaves of *G. bicolor* distinctively show reddish purple color on the abaxial side and green color on the adaxial side. The native geographic distribution of *G. bicolor *occurs in China, Taiwan, Myanmar, and Thailand. The *G. bicolor* is consumed as a culinary cooked vegetable and believed to confer a wide range of benefits such as anticancer, anti-inflammatory, and possibly antihypertensive effects. *G. bicolor* is also used for post-labor recovery, blood circulation improvement, treatment of dysmenorrhea, hemoptysis, and diabetes by the locals [1–3].

Although *G. bicolor* is widely used in Malaysia, there are no recorded data of locally grown *G. bicolor* species. Scientific studies on the use of locally grown *G. bicolor *to ensure its efficacy and nontoxicity have not been carried out. Several phytochemical and biological reports showed that *G. bicolor* cultivated in Japan, China, and Taiwan has antihyperglycemic effect [2, 4], anti-inflammation activity [5], and antioxidant capacity [6] and could induce apoptosis in HL60 leukemia cells [7]. Chemical constituents such as flavonols, anthocyanins, sesquiterpenes, terpenes, and megastigmane-type norisoprenoids had also been isolated from *G. bicolor *leaves [3, 8–10].

Oxidative stress plays an important role in carcinogenesis as free radicals could cause various modifications to biological macromolecules like DNA, protein, and lipid and could increase the risk of mutagenesis [11]. The present study aimed to evaluate the total phenolic content, antioxidant activity, cytotoxic effect, and acute oral toxicity of *G. bicolor *leaves. The crude methanol and its fractionated extracts (hexane, ethyl acetate, and water) were firstly prepared prior to the bioactivity assessments. Folin-Ciocalteu's method was used for the measurement of total phenolic content of extracts, while two established testing systems were carried out to evaluate the antioxidant capacity, namely, DPPH radical scavenging assay and metal chelating assay. These antioxidant assays have been widely used to determine the antioxidant capacities as these assays require standard equipments and deliver reproducible fast results. More than one antioxidant assays were performed in the present study because a single antioxidant assay is not sufficient to measure the various modes of action of antioxidants in a test sample. As *G. bicolor* is believed to have anticancer properties especially against colon cancer by the locals, the cytotoxic activities of *G. bicolor *extracts were investigated against four human colon adenocarcinoma cell lines with varying molecular characteristics (HT-29, HCT-15, SW480, and Caco-2), a human colon carcinoma cell line (HCT 116), a human breast adenocarcinoma cell line (MCF7), and a human normal colon cell line (CCD-18Co). There is limited information regarding the cytotoxic activity of *G*. *bicolor* on human cancer cell lines. To the best of our knowledge, this is the first report on the cytotoxic effect of *G. bicolor* on colon and breast adenocarcinoma cell lines. The cells induced with cytotoxic extract were then subjected to AO (acridine orange)/EB (ethidium bromide) staining and annexin-V/PI (propidium iodide) flow cytometry in order to assess the cell death and morphological changes. In view of the increasing popular consumption of *G. bicolor*, acute oral toxicity assay was carried out in the present study to ensure that *G. bicolor *is safe for human consumption. Hence, the resulting information will certainly provide some scientific validation on the traditional use of locally grown *G. bicolor *leaves.

## 2. Materials and Methods

### 2.1. Chemicals and Reagents

Gallic acid, BHA (butylated hydroxyanisole), DPPH (1,1-diphenyl-2-picrylhydrazyl), potassium ferricyanide, Folin-Ciocalteu's phenol reagent, AO (acridine orange), EB (ethidium bromide), MTT (methylthiazolyldiphenyl-tetrazolium bromide), RPMI 1640 medium, McCoy's 5A medium, EMEM (Eagle's minimum essential medium), sodium bicarbonate, *cis*-platin, carboxymethyl cellulose, EDTA (ethylenediaminetetraacetic acid), and DMSO (dimethyl sulfoxide) were purchased from Sigma-Aldrich Company. Fetal bovine serum, penicillin/streptomycin (100X), amphotericin B (250 *μ*g/mL), and sodium pyruvate (100 mM) were from PAA Laboratories. Methanol, hexane, and ethyl acetate were purchased from Fisher Scientific Company. FITC Annexin V Apoptosis Detection Kit I was purchased from BD Biosciences Company.

### 2.2. Plant Sample Collection and Identification

Fresh leaves of *G. bicolor* were collected from Seremban, Negeri Sembilan, Malaysia, in February 2011. The identification of the plant species was done by Dr. Yong Kien Thai at the Institute of Biological Sciences, Faculty of Science, University of Malaya, Malaysia. A voucher specimen (herbarium no. KLU47744) was deposited at the herbarium of the Institute of Biological Sciences, Faculty of Science, University of Malaya, Kuala Lumpur, Malaysia.

### 2.3. Preparation of Extracts

Briefly, the fresh leaves of *G. bicolor* (8049.20 g) were washed, dried, and ground to fine powder (768.70 g, 9.55%). The powder was extracted with methanol at room temperature yielding a dark green crude methanol extract (76.10 g, 9.90%). The crude methanol extract (61.10 g) was further extracted with hexane to obtain hexane-soluble extract (23.40 g, 38.30%) and hexane-insoluble residue. Hexane-insoluble residue was further partitioned with ethyl acetate-water (1 : 1) to yield ethyl acetate (1.40 g, 2.29%) and water (11.9 g, 19.48%) extracts. The percentage yield of fractionated extracts was based on the weight of crude methanol extract (61.10 g) used for fractionation. All of the extracts (methanol, hexane, ethyl acetate, and water) were kept in the dark at 4°C for no more than one week prior to evaluation of total phenolic content, antioxidant effect, cytotoxic activity, and acute oral toxicity.

### 2.4. Determination of Total Phenolic Content

The phenolic content of *G. bicolor* extracts was determined by Folin-Ciocalteu's method that was modified to accommodate the 96-well plate as described previously by Sulaiman and Ooi [12]. Briefly, 25 *μ*L of Folin-Ciocalteu's reagent was firstly added to 10 *μ*L of extract solution (concentrations ranged 4–20 mg/mL) in each well of 96-well plate. The mixture was then incubated at room temperature for five minutes before adding 25 *μ*L of 20% (w/v) sodium carbonate to the mixture. This was then followed by addition of distilled water to make a final volume of 200 *μ*L per well. The reaction mixture was further incubated for 30 minutes at room temperature before the absorbance reading taken at 760 nm using a microplate reader (Thermo Scientific Multiskan GO). A standard curve was plotted using gallic acid (0–1000 mg/L). BHA was used as the positive reference standard in the present study. All of the extracts and positive reference standard were assayed in triplicate. The results were expressed as milligram of gallic acid equivalents per gram of extract (mg of GAEs/g of extract).

### 2.5. DPPH Radical Scavenging Activity

DPPH radical scavenging activity of *G. bicolor* extracts was measured according to the method described by Sulaiman and Ooi [12]. Briefly, 150 *μ*L of DPPH solution (0.3 mM) was added to 50 *μ*L of extract (at various concentrations) in each well of 96-well plate before incubation for 30 minutes at room temperature. As for the blank, 50 *μ*L of distilled water or methanol was added to the DPPH solution instead of the extract. The absorbance value was taken at 515 nm using a microplate reader (Thermo Scientific Multiskan GO) after incubation. BHA was used as positive reference standard in the present study. All of the extracts and positive reference standard were assayed in triplicate. The scavenging ability of the extract was expressed as IC_50_ value, which is the concentration at which 50% of DPPH radicals were scavenged. The IC_50_ value was obtained by extrapolating from the graph of DPPH radical scavenging activity (%) versus concentration of extract.

### 2.6. Metal Chelating Assay

The metal chelating assay was performed in 96-well plate based on protocol described by Sulaiman and Ooi [12]. Briefly, 50 *μ*L of extract (at various concentrations) was incubated with 5 *μ*L of ferrous chloride hexahydrate (2 mM) and 130 *μ*L of deionized water in each well of a 96-well plate for 5 minutes at room temperature. The reaction was initiated by the addition of 15 *μ*L ferrozine (5 mM). After reaction mixture reached equilibrium, the absorbance value was measured at 562 nm using a microplate reader (Thermo Scientific Multiskan GO). EDTA was used as positive reference standard for the experiment. All of the extracts and positive reference standard were assayed in triplicate. The chelating effect of the extracts was expressed as IC_50_, which is the concentration at which 50% of metal ions were chelated. The lower IC_50_ value indicates the stronger metal chelating ability of the extract.

### 2.7. MTT Cytotoxicity Assay

Human colon adenocarcinoma (HT-29, HCT-15, SW480, and Caco-2), colon carcinoma (HCT 116), breast adenocarcinoma (MCF7), and human normal colon (CCD-18Co) cell lines were purchased from American Type Culture Collection (ATCC, USA). HCT-15, SW480, and MCF7 cells were maintained in RPMI 1640 medium; HT-29 and HCT 116 cells in McCoy's 5A medium; Caco-2 and CCD-18Co cells in EMEM medium. All media were supplemented with 10% fetal bovine serum, 1% penicillin/streptomycin (100X), and 0.5% amphotericin B except for EMEM medium that was supplemented with additional 1% sodium pyruvate. Caco-2 cells were maintained in EMEM medium which was supplemented with 20% fetal bovine serum instead of 10%. The cells were cultured at 37°C in CO_2_ incubator.

The MTT cytotoxicity assay was carried out as described previously by Mosmann [13]. All of the extracts were firstly dissolved in DMSO (with the exception of water extract which was dissolved in distilled water) to form stock solutions of 20 mg/mL before testing. Briefly, cells were seeded into 96-well plate for 24 hours before treatment with various concentrations (1, 10, 25, 50, 75, and 100 *μ*g/mL) of the extract. The final concentration of DMSO in each well was 0.5%. Untreated cells were used as negative controls. Following 24, 48, and 72 hours of incubation, 20 *μ*L of MTT (5 mg/mL) was added into each well and further incubated for another four hours. The medium was then removed and replaced with DMSO. The absorbance was measured at 570 nm with 650 nm as background using a microplate reader (Thermo Scientific Multiskan GO). All of the extracts were assayed in triplicate. *Cis*-platin was used as a positive reference standard. IC_50_ value is the concentration of extract or positive reference standard that inhibits 50% of the cells growth.

### 2.8. AO/EB Double Staining

Briefly, HCT 116 cells were seeded into 6-well plate (1 × 10^5^ cells/well) for 24 hours before treatment with 20 *μ*g/mL of cytotoxic ethyl acetate extract. Untreated cells were used as negative controls. After incubation of 24 hours, 160 *μ*L of AO/EB solution (one part of 100 *μ*g/mL AO and one part of 100 *μ*g/mL EB in PBS) was loaded into each well which contained 2 mL of medium. The cells were immediately visualised under inverted fluorescence *microscope* (Olympus IX73) with blue excitation mirror unit at 200x magnification. *Cis*-platin (20 *μ*g/mL) was used as positive reference in the present study.

### 2.9. Annexin-V/PI Flow Cytometry

Briefly, HCT 116 cells (2 × 10^5^ cells) were seeded in 60 mm petri dish. After 24 hours, HCT 116 cells were treated with 20 *μ*g/mL of cytotoxic ethyl acetate extract. Untreated cells were used as negative controls. After 24 hours, cells were harvested and stained with FITC annexin-V and PI following instruction of the FITC Annexin V Apoptosis Detection Kit I. Cells treated with 20 *μ*g/mL of c*is*-platin were used as positive controls. A total of 10,000 cells were analyzed by flow cytometer (BD FACSCanto II) using FACSDiva software.

### 2.10. Acute Oral Toxicity

The study was performed on healthy male Sprague-Dawley rats (aging 8–12 weeks; mean body weight 209 g) which were obtained from the Laboratory Animal Center, Faculty of Medicine, University of Malaya. The weight variation in the rats did not exceed ±20% SD of the mean weight. The animal research protocol was approved by the Institutional Animal Care and Use Committee, University of Malaya (UM IACUC) before commencing the study (ethical no. ISB/29/06/2012/SKS (R)).

The acute oral toxicity of *G. bicolor *was conducted using the procedure described by OECD guideline 423 [14] with some modification. The crude methanol extract was firstly suspended in 0.3% carboxymethyl cellulose suspension (vehicle). The male rats were assigned to three treatment groups and one control group, with three rats for each group. The treatment groups were dosed at 300, 2000 and 5000 mg of crude methanol extract per kilogram of body weight, while the control group was administered with vehicle only. Rats were housed in stainless steel, wire-mesh cages in an experimental animal room (ventilated, 25°C, 50%–60% humidity, and 12-hour light/dark cycle). The rats had free access to water and food and were acclimatized to the room condition for five days before starting the experiment. The rats were fasted for 12 hours prior to dosing (access to water only). After fasting, the body weights of rats were recorded. The rats were dosed using a stainless steel ball-tipped gavage needle attached to an appropriate syringe. The volume of administration is 1 mL/100 g of body weight. After dosing, food was withheld for four hours before providing it to the rats. All rats were observed for mortality, sign of toxicity, and behavioral changes at four hours after dosing and daily for 14 days. Individual weights were recorded from day 1 to day 14. The experiment was performed twice for each dose.

### 2.11. Statistical Analysis

The antioxidant data in the present study were subjected to one-way analysis of variance (ANOVA), and the significance of the difference between the means was determined by Duncan's multiple-range tests at 95% least significant difference (*P* < 0.05). The IC_50_ values for cytotoxic activity were obtained by nonlinear regression using GraphPad Prism statistical software.

## 3. Results and Discussion

### 3.1. Total Phenolic Content of *G. bicolor* Extracts

Phenolic compounds such as phenolic acids, flavonoids, and tannins are the major determinants of antioxidant potentials in foods, and they could be a natural source of antioxidants, antimutagenic, and antitumour activities [15]. In the present study, Folin-Ciocalteu's method was used to determine the total phenolic content of *G. bicolor* extracts as it is rapid, reproducible, simple, and convenient. The absorbance value of *G. bicolor *extracts after subtraction of control (*y*) was translated into total phenolic content (mg/L of gallic acid equivalents (GAEs)) using the gallic acid calibration plot with the following formula: total phenolic content = (*y* − 0.0496)/0.001; *R* ^2^ = 0.9855. Total phenolic content of ethyl acetate extract was significantly higher than those of other extracts (*P* < 0.05). The total phenolic content of *G. bicolor* extracts in descending order was ethyl acetate > hexane > methanol > water, as shown in Table 1, with the values varying from 0.28 to 10.87 mg of GAEs/g of extract. The ethyl acetate extract has the highest phenolic content with 10.87 mg of GAE/g of extract, followed by hexane (0.91 mg of GAE/g of extract), methanol (0.75 mg of GAE/g of extract), and water (0.28 mg of GAE/g of extract) extracts. Hexane extract usually contains high level of oil, wax, and chlorophyll, while water extract contains high sugar content. This could be the reason for lower phenolic content in both of the extracts. Phenolic compounds such as quercetin, kaempferol, quercitrin, isoquercitrin, rutin, and three poly-acylated anthocyanins (rubrocinerarin, bicolnin, and bicolmalonin) had been identified in *G. bicolor* leaves in previous studies reported by Lu et al. [3] and Shimizu et al. [9]. These phenolic compounds may be present in the ethyl acetate extract and contribute to the high phenolic content of ethyl acetate extract in the current study.

### 3.2. DPPH Radical Scavenging Activity of *G. bicolor *Extracts

Radical scavenging activity is very important due to the deleterious role of free radicals in food and biological systems [16]. DPPH radical scavenging assay is a discoloration assay, which evaluates the absorbance decrease at 515–528 nm produced by the addition of the antioxidant to a DPPH solution in ethanol or methanol. It is sensitive and easy to perform, and it offers a rapid way to screen radical scavenging activity of the tested samples. The lower IC_50_ value indicates stronger ability of the extract to act as DPPH scavenger as lesser scavengers were required to achieve 50% scavenging reaction.

In the present study, *G. bicolor* extracts were investigated through the free radical scavenging activity *via* their reactions with the stable DPPH radicals (Table 2). Ethyl acetate extract appeared to be the major contributor for antioxidant capacity of *G. bicolor* in DPPH assay as DPPH scavenging activity of ethyl acetate extract was significantly higher than those of other extracts (*P* < 0.05). The descending order of DPPH scavenging activity of *G. bicolor* extracts was ethyl acetate > methanol > hexane > water. The ethyl acetate extract exhibited the lowest IC_50_ value (0.53 mg/mL) followed by methanol, hexane, and water extracts with IC_50_ values of 4.93, 11.15, and 13.57 mg/mL, respectively. This indicates that the ethyl acetate extract may contain antioxidative substances which have hydrogen-donating activity to scavenge DPPH radicals and are able to terminate the chain reaction of free radicals.

### 3.3. Metal Chelating Activity of *G. bicolor* Extracts

The metal ion chelating ability plays a significant role in the antioxidant mechanism because it prevents oxyradical generation and the consequent oxidative damage [17]. In the present study, the metal chelating ability of *G. bicolor* extracts was investigated by assessing the ability of the antioxidants to compete with the indicator ferrozine to complex with ferrous ion (Fe^2+^) in solution. Metal chelating activity of ethyl acetate extract was significantly higher than those of other extracts (*P* < 0.05). The metal chelating activity of *G. bicolor *extracts in descending order was ethyl acetate > methanol > hexane > water, as shown in Table 2. The methanol, hexane, and ethyl acetate extracts showed comparable metal chelating activity with IC_50_ values in the range of 3.80–4.90 mg/mL, while water extract on the other hand showed inhibitory activity only at 28.37 mg/mL. All of the extracts showed low metal chelating capability as compared to EDTA, which was the positive reference standard. This could be possibly due to lack of chemical compounds that could act as metal chelators in the *G. bicolor* extracts.

### 3.4. Cytotoxic Activities of *G. bicolor* Extracts

It is generally known that the genetic background of cell lines could affect the sensitivity of anticancer agent. In the present study, the cytotoxic activity of *G. bicolor* extracts was evaluated against four human colon adenocarcinoma cell lines with varying molecular characteristics (HT-29, HCT-15, SW480, and Caco-2), one colon carcinoma cell line (HCT 116), and one human breast adenocarcinoma cell line (MCF7) using the MTT cytotoxicity assay which measures the mitochondrial activity in viable cells. The human normal colon cell line (CCD-18Co) was used to determine the specificity for cancerous cells. According to the United States National Cancer Institute Plant Screening Program, a plant extract is considered to possess cytotoxicity if the IC_50_ value is 20 *μ*g/mL or less after incubation between 48 and 72 hours [18].

The results of cytotoxicity screening of *G. bicolor* extracts against the selected human cell lines, expressed as IC_50_ value averaged from three experiments, are summarized in Table 3. All of the extracts did not show any cytotoxic effect (with IC_50_ values > 100 *μ*g/mL against all the tested human cell lines), except that ethyl acetate extract with cytotoxic sensitivity differed between the various human cancer cell lines. Among the tested cells, cytotoxicity of ethyl acetate extract was found to be most active against HCT 116 and HCT-15 cells, with the IC_50_ values of 16.0 and 12.8 *μ*g/mL after 24 hours of incubation; 20.9 and 18.7 *μ*g/mL after 48 hours of incubation, respectively. HCT 116 and HCT-15 are the colon cancer cell lines which have wild-type p53, and both are MMR-deficient, while HT-29, SW480, and Caco-2 cells have mutant p53 and are MMR-proficient [19]. Based on the current result, it was speculated that the cytotoxicity of ethyl acetate extract was mediated by wild-type p53 and MMR-deficiency of colon cell lines. On the other hand, MCF7 cells which also expressed wild-type p53 [20] showed lower cytotoxicity than HCT 116 and HCT-15 cells; this could be due to different tissue origins of cells.

Generally, the tested human cancer cell lines exhibited time-dependent response, by which IC_50_ values obtained were increased over time (24, 48, and 72 hours). The cytotoxic effect of ethyl acetate extract was reduced when the treatment duration increased. The tested carcinoma cell lines seem to be able to overcome the treatment inhibitory effect. This can be explained by the possibility that the ethyl acetate extract may contain some chemical constituents which may repair the cell mechanism in order to increase cell growth. Another explanation could be due to the stability of the active chemical constituents in ethyl acetate extract by which the chemical constituents may be degraded over time.

As shown in Table 3, the cytotoxic effect of ethyl acetate extract on normal colon cells (CCD-18Co) was not detected (IC_50_ > 100 *μ*g/mL). This indicated that ethyl acetate extract was selectively toxic against cancerous cell lines. Thus, the active chemical constituents in the ethyl acetate extract may lead to valuable constituents that may have the ability to kill cancerous cells but exert no damage to normal cells. The cytotoxic effect of ethyl acetate extract may be due to the presence of anthocyanins and flavonols which had been identified in previous studies [3, 7, 9]. Anthocyanins are the chemical constituents which contribute to the purple color of *G. bicolor *leaves [9] and are reported to have antiproliferation effect on variety of carcinoma cell lines [21]. Few flavonols such as quercetin and kaempferol which had been isolated from *G. bicolor* leaves by Lu et al. [3] were reported to have the ability to suppress the growth of HT-29, SW480, and HCT 116 cancer cells [22–24]. As compared to other *Gynura* species, cytotoxic cerebroside (gynuraoside) which had been identified in ethyl acetate extract of *G. divaricata* [25] may be present in *G. bicolor* as well and thus contributed to the cytotoxic activity of the *G. bicolor* ethyl acetate extract.

### 3.5. Apoptosis and Necrosis Evaluation by AO/EB Staining and Annexin-V/PI Flow Cytometry

In cell death induction, apoptosis is usually more desired than necrosis as apoptosis is a normal well-regulated process and does not often result in inflammation, while necrotic cell death is caused by external injury and almost always results in inflammation [26]. However, necrotic cell death can be useful to manage apoptosis-resistant tumors [26, 27].

In the present study, AO/EB staining was used to assess the type of cell death and morphological changes induced by cytotoxic ethyl acetate extract of *G. bicolor*. AO (acridine orange) can penetrate viable and nonviable cells and makes the nuclei appear green, while nonviable cells which lost the membrane integrity will take up EB (ethidium bromide) that makes the nuclei appear red. Hence, viable cells will have green organized structure nuclei; early apoptotic cells will have green nuclei with condensed or fragmented chromatin; late apoptotic cells will have orange to red nuclei with condensed or fragmented chromatin (EB overwhelms AO); necrotic cells will show orange to red organized structure nuclei.

**Figure 1:**
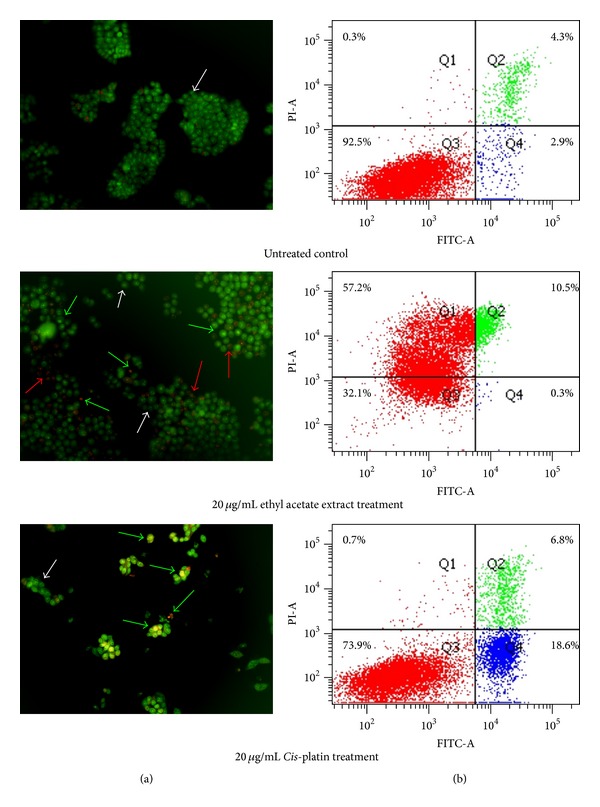
Cell death evaluation by AO/EB staining and annexin-V/PI flow cytometry. (a) HCT 116 cells stained with AO/EB; white arrows indicate live cells, green arrows indicate apoptotic cells, and red arrows indicate necrotic cells. Magnification is 200x. (b) HCT 116 cells stained with FITC annexin-V/PI. Experiments were repeated twice with similar outcome.

As shown in Figure 1(a), apoptotic and necrotic HCT 116 cells were detected when treated with ethyl acetate extract of *G. bicolor*. The ethyl acetate extract-treated HCT 116 cells were rounding up compared with untreated control cells. Chromatin condensation is one of the hallmarks of apoptosis. Green and red nuclei with condensed chromatin were observed in HCT 116 cells after 20 *μ*g/mL of ethyl acetate extract treatment, indicating that ethyl acetate could induce HCT 116 cells to undergo early and late apoptosis, whilst red nuclei with noncondensed chromatin observed in Figure 1(a) (ethyl acetate treatment) indicated the necrotic cell death in HCT 116 cells as lack of chromatin condensation.

In order to further asses and confirm the type of cell death induced by cytotoxic ethyl acetate extract of *G. bicolor*, flow cytometry was used to analyze FITC annexin-V and PI stained cells as it can quantitatively determine the percentage of cells within a population that are undergoing early and late apoptosis or necrosis. During early apoptosis, PS (phosphatidylserine) is translocated from inner to outer leaflet of plasma membrane. The exposed PS could be detected by FITC conjugated annexin-V. In the event of late apoptosis and necrosis, loss of membrane integrity could be detected by PI.

According to Figure 1(b), untreated HCT 116 cells showed low or negative staining with both annexin-V and PI (Q3; annexin-V−/PI−), indicating viable cells. After treatment with 20 *μ*g/mL of ethyl acetate extract, HCT 116 cells demonstrated an increase of necrotic cells (Q1; annexin-V−/PI+) from 0.3% (untreated control) to 57.2%. Cells stained with both annexin-V and PI (Q2; annexin-V+/PI+), which represented later stage of apoptosis, were also found to increase from 4.3% (untreated control) to 10.5% after treatment with the ethyl acetate extract. Taken together, the results clearly showed that the ethyl acetate extract appeared to induce late apoptosis and necrosis in HCT 116 cells.

### 3.6. Acute Oral Toxicity of *G. bicolor* Methanol Extract

Acute oral toxicity test was carried out in the present study to evaluate the safety of *G. bicolor* methanol extract as human consumption. As shown in Table 3, the *G. bicolor* extracts did not show cytotoxic activity against the normal cell line (IC_50_ > 100 *μ*g/mL). If this occurs *in vivo* as well, the use of *G. bicolor* as natural remedies would have scientific support. Throughout the 14 days of observation period after oral administration, there were no deaths reported and no changes observed on the outer appearance (skin, fur, eyes, and mucous membranes) of treated rats which were dosed at 300, 2000, and 5000 mg of crude methanol extract per kilogram of body weight. The treated rats also did not show any sign of toxicity (loss of appetite, vomiting, constipation, diarrhoea, dysphagia, hematemesis, hematochezia, tremors, convulsions, salivation, and coma) or behavioral changes (hyperactivity and hypoactivity). The overall condition of treated rats was similar to that of control rats.

As shown in Table 4, the mean body weight of all treatment groups gradually increased over the observation period. According to OECD guideline 423 [14], the weight variations in rats are acceptable if they did not exceed ±20% SD of the mean weight. *G. bicolor* methanol extract was classified in category 5 according to Globally Harmonized Classification System for Chemical Substances and Mixtures (GHS) of OECD as test sample in category 5 is of relatively low acute toxicity and the expected LD_50_ is more than 5000 mg/kg. This is in agreement with Kennedy et al. [28] who stated that test sample with LD_50_ more than 5000 mg/kg is considered as nontoxic. The current result of acute oral toxicity is in agreement with that of no *in vitro* toxicity against the normal cell line and confirmed the safety of the *G. bicolor* for consumption. As compared to other *Gynura* species, the ethanol extract of *G. procumbens* also showed no toxicity to experimental rats dosed at 2 g/kg and 5 g/kg [29].

## 4. Conclusion

The present research findings have evidently met the objectives of the study. The ethyl acetate extract of *G. bicolor *with the highest total phenolic content among the extracts generally showed the strong ability in scavenging DPPH radicals and metal chelating activity. The current study had also clearly demonstrated for the first time that the ethyl acetate extract of *G. bicolor *strongly possessed cytotoxic activity against the human cancer cell lines and induced apoptotic and necrotic cell death, especially towards the HCT 116 and HCT-15 colon cancer cells. The acute oral toxicity indicated that *G. bicolor* has negligible level of toxicity when administered orally and has been regarded as safe in experimental rats. Further work is now on the way to investigate the bioactive chemical constituents in ethyl acetate extract and mechanism of cell death in order to provide more convincing evidence on the traditional usage of *G. bicolor.*

## Figures and Tables

**Table 1:** Total phenolic content of *G. bicolor* extracts.

Extract	Concentration of total phenolics (mg of GAEs/g of extract)
Methanol	0.75 ± 0.02^b^
Hexane	0.91 ± 0.04^b^
Ethyl acetate	10.87 ± 0.22^c^
Water	0.28 ± 0.02^a^
BHA*	24.70 ± 0.07^d^

*Positive reference standard; GAEs: gallic acid equivalents; values are expressed as mean ± standard deviation (*n* = 3); means with different letters (a–d) in the same column were significantly different (*P* < 0.05, ANOVA).

**Table 2:** The IC_50_ values of *G. bicolor* extracts in DPPH radical scavenging activity and metal chelating assay.

Extract	IC_50_ value (mg/mL)	
	DPPH radical scavenging activity	Metal chelating assay
Methanol	4.93 ± 0.15^c^	4.10 ± 0.10^b^
Hexane	11.15 ± 0.21^d^	4.90 ± 0^c^
Ethyl acetate	0.53 ± 0.01^b^	3.80 ± 0.10^b^
Water	13.57 ± 0.23^e^	28.37 ± 0.40^d^
BHA*	0.03 ± 0^a^	—
EDTA*	—	0.04 ± 0^a^

*Positive reference standard. Values are expressed as mean ± standard deviation (*n* = 3); means with different letters (a–e) in the same column were significantly different (*P* < 0.05, ANOVA).

**Table 3:** Cytotoxic activity (IC_50_ values) of *G*. *bicolor* extracts against selected human cell lines.

Extract	Treatment duration (hour)	Cytotoxicity (IC_50_) in *µ*g/mL						
		HT-29	HCT 116	HCT-15	SW480	Caco-2	MCF7	CCD-18Co
Methanol	24	>100	>100	>100	>100	>100	>100	>100
	48	>100	>100	>100	>100	>100	>100	>100
	72	>100	>100	>100	>100	>100	>100	>100

Hexane	24	>100	>100	>100	>100	>100	>100	>100
	48	>100	>100	>100	>100	>100	>100	>100
	72	>100	>100	>100	>100	>100	>100	>100

Ethyl acetate	24	39.7 ± 1.7	16.0 ± 4.5	12.8 ± 5.3	26.3 ± 1.2	32.7 ± 1.5	36.5 ± 3.4	>100
	48	49.5 ± 8.6	20.9 ± 0.2	18.7 ± 1.9	31.7 ± 5.7	46.3 ± 8.7	34.8 ± 0.8	>100
	72	37.3 ± 2.5	29.2 ± 0.9	21.2 ± 1.2	47.8 ± 3.6	55.2 ± 6.4	46.3 ± 4.2	>100

Water	24	>100	>100	>100	>100	>100	>100	>100
	48	>100	>100	>100	>100	>100	>100	>100
	72	>100	>100	>100	>100	>100	>100	>100

*Cis*-platin*	24	>12.5	12.0 ± 0.7	6.2 ± 0.4	>12.5	>12.5	11.2 ± 0.5	>12.5
	48	10.1 ± 0.2	4.0 ± 0.3	3.9 ± 0.2	6.8 ± 0.5	4.3 ± 0.4	4.2 ± 0.4	>12.5
	72	6.4 ± 0.6	2.9 ± 0.1	1.7 ± 0.4	3.2 ± 0.6	1.9 ± 0.2	2.7 ± 0.3	>12.5

*Positive reference standard. Values are expressed as mean ± standard deviation (*n* = 3).

**Table 4:** The effect of methanol extract of *G. bicolor *on rat body weight.

Treatment dose (mg/kg)	Body weight (g)													
	Day 1	Day 2	Day 3	Day 4	Day 5	Day 6	Day 7	Day 8	Day 9	Day 10	Day 11	Day 12	Day 13	Day 14
Control	183 ± 6	183 ± 10	187 ± 12	188 ± 10	192 ± 16	197 ± 16	195 ± 13	197 ± 12	198 ± 14	200 ± 17	205 ± 23	210 ± 20	212 ± 18	215 ± 18
300	218 ± 17	223 ± 16	223 ± 17	227 ± 16	230 ± 16	233 ± 18	233 ± 16	240 ± 18	243 ± 18	248 ± 19	249 ± 18	253 ± 17	256 ± 20	259 ± 21
2000	221 ± 24	223 ± 23	226 ± 24	228 ± 22	230 ± 23	231 ± 21	234 ± 22	237 ± 20	238 ± 22	241 ± 24	244 ± 23	246 ± 24	249 ± 25	252 ± 25
5000	200 ± 28	203 ± 27	204 ± 28	208 ± 26	210 ± 28	213 ± 28	218 ± 24	220 ± 25	223 ± 25	228 ± 23	230 ± 24	233 ± 24	238 ± 24	240 ± 25

Values are expressed as mean ± standard deviation (*n* = 6).
